# Methodological Selection of Optimal Features for Object Classification Based on Stereovision System

**DOI:** 10.3390/s24123941

**Published:** 2024-06-18

**Authors:** Rafał Tkaczyk, Grzegorz Madejski, Dawid Gradolewski, Damian Dziak, Wlodek J. Kulesza

**Affiliations:** 1 Bioseco S.A., Budowlanych 68, 80-298 Gdansk, Poland; grzegorz.madejski@ug.edu.pl (G.M.); dawid.gradolewski@bioseco.com (D.G.); damian.dziak@bioseco.com (D.D.); 2 Institute of Informatics, Faculty of Mathematics, Physics and Informatics, University of Gdańsk, 80-308 Gdansk, Poland; 3 Department of Mathematics and Natural Sciences, Blekinge Institute of Technology, 371 79 Karlskrona, Sweden; wlodek.kulesza@bth.se

**Keywords:** avifauna classification, æms, feature extraction, IoT, nature conservation, smart sensing, wildlife hazard management, wind farms

## Abstract

With the expansion of green energy, more and more data show that wind turbines can pose a significant threat to some endangered bird species. The birds of prey are more frequently exposed to collision risk with the wind turbine blades due to their unique flight path patterns. This paper shows how data from a stereovision system can be used for an efficient classification of detected objects. A method for distinguishing endangered birds from common birds and other flying objects has been developed and tested. The research focused on the selection of a suitable feature extraction methodology. Both motion and visual features are extracted from the Bioseco BPS system and retested using a correlation-based and a wrapper-type approach with genetic algorithms (GAs). With optimal features and fine-tuned classifiers, birds can be distinguished from aeroplanes with a 98.6% recall and 97% accuracy, whereas endangered birds are delimited from common ones with 93.5% recall and 77.2% accuracy.

## 1. Introduction

In the wake of expanding human infrastructures, birds are more and more frequently exposed to collision risk with wind turbines [1,2,3,4,5,6] and aeroplanes [7]. Much effort has been recently made to develop solutions that ensure comprehensive protection of birds and meet the challenging requirements of environmental authorities [8,9]. The bird protection system (BPS) uses stereovision that provides precise 3D localisation to prevent collisions with wind turbine blades [10,11]. However, reliable species classification can improve deterrence systems and reduce wind turbine stoppages [12,13]. Hence, a reliable real-time bird species/genus/family classification algorithm recently became an emerging research topic [11].

Safety applications implicate the requirement of a high classification accuracy, which, according to the German Competence Center for Nature Conservation and Energy Transition (Kompetenzzentrum Naturschutz und Energiewende, KNE), needs to exceed 90% in sensitive areas [14]. This, combined with the required detection range, as far as 500 meters in 360 ° around the wind turbine and the harsh environmental impact on the measurement data, makes the development of a robust classification algorithm a nontrivial task.

We propose to apply a two-stage binary classification approach. In the first stage, birds are distinguished from other flying objects, such as aeroplanes. In the second stage, protected big birds of prey are distinguished from other birds. In this paper, we investigate how to optimise the selection of the features to ensure accurate classification. We test state-of-the-art classifiers such as neural network (NN), decision tree (DT), random forest (RF), and support vector machine (SVM). The optimisation is based on two approaches: the correlation coefficient method and genetic algorithms (GAs).

## 2. Background Knowledge and Related Works

The works that are most relevant to the presented interdisciplinary research are those related to using machine learning methods for flying object classification with a main focus on feature extraction.

### 2.1. Flying Object Features

Flying objects’ feature extraction can be approached from ornithological and technological perspectives, while our interest is in the latter. Three common classes of features are used in flying object classification.

1. **Sound:** The analysis of frequency, duration, and volume of bird vocalizations collected by microphones through passive acoustic monitoring [15,16].
2. **Morphology:** The analysis of the size of a bird, its shape (beak, wings, etc.), or plumage colour based on the video or image recordings of optical sensors [16].
3. **Motion and behavioural patterns:** The analysis of a bird’s (or other object’s) flight trajectories and their features, including turning angle, curvature, velocity, acceleration, or periodic oscillation due to wing flapping. The data can be gathered using various types of sensors: optical [17], radar [18], or accelerometer [19].

Since the wind farm and airport environments (in terms of sound) are noise-contaminated, this study focuses on morphological, motion, and behavioural patterns.

### 2.2. Optimal Feature Selection Methods

The extracted features must be evaluated to create their optimal set for classification. For this purpose, when researching the literature, two basic approaches can be found, i.e., statistical and wrapper methods.

Correlation between features and class variables as a criterion for feature selection is a statistical approach that uses features with higher correlation for classification. In a study by Atanbori et al. [20], this selection method was used for an RF classifier, and it improved the bird classification accuracy from 83% to 90%.

The wrapper method for optimal feature selection uses a machine learning model to evaluate the selection and adjust the selected features to improve the classification performance, and the GA belongs to this category. It randomly generates a set of input features and then iteratively evaluates solutions to converge to the optimal feature set. Qian [21] used a GA method combined with mutual information measures to select optimal features from audio and image databases. The classification scores were higher than those of other selection methods. Putrada and Prabowo [22] proved that GA is suitable for selecting optimal features to enhance the performance of the aeroplane-bird classifier. The area under the curve *AUC* score improved from 87.8% to 88.9%, and the *recall* improved from 77.3% to 80%.

### 2.3. Flying Object Classifiers

The comprehensive review of machine learning methods by Principato et al. [16] includes updated information on bird-related classification approaches: preprocessing techniques (image augmentation and audio signal transforms), classification algorithms (convolutional neural network, CNN; K-nearest neighbours, kNN; RF), or classifier evaluation metrics (accuracy and precision). We supplemented the overview with a few scientific papers that were not mentioned in the survey. We focus on four classifiers used in this study: NN, DT, RF, and SVM. We also analyse evaluation metrics suitable for bird classification.

In [23,24], a NN was used for bird recognition based on birds’ voices. In [18], based on micro-Doppler spectrogram images, a CNN was used to classify drones, birds, clutter, and noise, with a 94% accuracy. A hybrid CNN-RNN model ensured an accuracy of 99% in classifying birds and drones based on synthetically generated (emulated) flight trajectories [25].

A DT is a transparent model that selects classification features and is efficient for queries. Qiao et al. [26] used a hybrid of the DT and SVM classifiers to recognise 15 species of birds based on their morphological features, with an 84% accuracy. A DT was also accurate in 95% of cases in identifying the type of kestrel flight behaviour, such as hovering, flapping, etc., based on features gathered by accelerometers placed on birds [19].

An RF is an ensemble classifier consisting of a collection of trees that vote on the class of the given sample, and it is considered a robust model that does not overfit easily. RF was used to classify 13 species of birds based on their motion features and was the best of the tested classifiers (SVM, DT, naive Bayes), with an accuracy of 90% [20]. RF also outperformed the K-nearest neighbour (KNN) method and logistic boost classifiers in the identification of seven seabird species using images reaching 86% in *precision*, *recall* and $F_{1}$ *measure* [27]. It was used to classify 14 Australian birds according to their sound recordings with 89% accuracy [28].

SVMs are classifiers that find the hyperplane that best separates different classes in the feature space, maximising the margin between the closest points of the classes. They are solid classifiers that are commonly used in flying object recognition. Atanbori et al. [29] classified seven species of birds based on their colour and motion features using SVMs and the naive Bayes method, with an accuracy of 89% and 92%, respectively. For binary classification of toucan and snowy owl based on colour features, SVMs performed with an accuracy of 98% [30]. SVMs were also tested for bird, drone, and kite recognition based on flight trajectory features with an accuracy of 85% [17].

Based on the given task and end-user requirements, the classifying model not only has to be reliable but also tested with appropriate measures. The *accuracy* as a separate score does not capture some problem intricacies, such as different error weights of type I (false positive, FP) and II (false negative, FN). Therefore, many studies apply other methods: *AUC*, *precision*, *recall*, or $F_{1}$ *measure* [10,22,27]. Apart from *accuracy*, this study uses *recall* as a crucial measure. This minimises the FN errors (endangered birds misclassified as other types of birds or birds misclassified as aeroplanes).

## 3. Problem Statement, Objectives, and Main Contributions

As the overview of related works shows, the classification of flying objects, especially birds, is of interest to researchers in different fields for diverse applications. We focus on bird classification systems based on stereovision for long-distance observations. The data from such systems can be challenging due to different types of interference and quantization errors [7,10].

This paper mainly aims to analyse different methods for optimal feature selection. We seek to determine whether the correlation coefficients method or GA provides a more efficient subset of features in terms of classification performance than the whole set of features. The performance was measured using *accuracy* and *recall* scores. The selection methods were examined based on four different classifiers: multi-layer perceptrons NN, DT, RF, and SVM.

**Figure 1:**
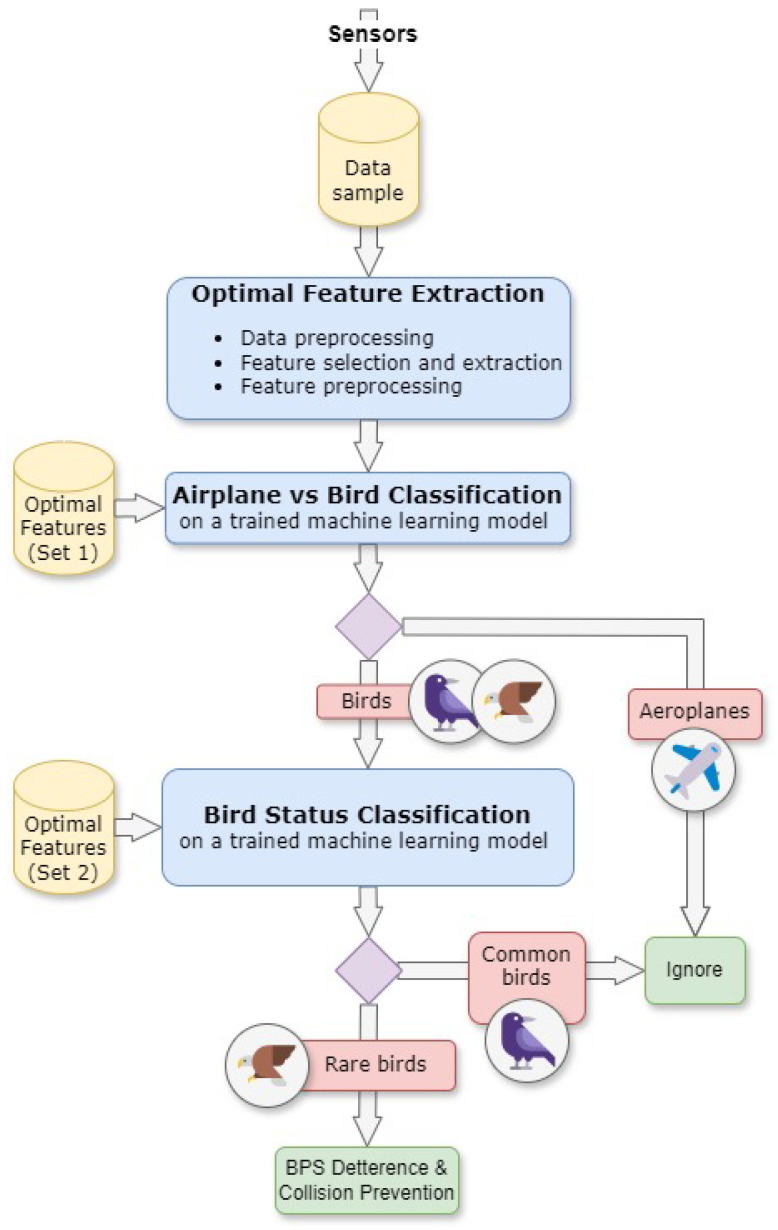
A block diagram of a real-time system for classifying aeroplanes, rare birds, and common birds.

Moreover, we investigated if a two-stage classification method has a performance that is sufficient for recognising a specific group of objects, as shown in Figure 1. In the first stage, the system separates avifauna from aeroplanes, and the hypothesis is that the classifiers can reach an accuracy of 95% and a recall of 97%. In the second stage, endangered birds (big raptors from *Accipitriformes* order: *Buteo buteo*, *Milvus milvus*, *Haliaeetus albicilla*) are distinguished from common birds. This classification should have at least 75% accuracy and 90% recall to be of practical use [8,9].

Finally, the study gives insights into feature selection. We investigate whether preselected features are sufficient for accurate classification and which optimal features selected by the algorithms are most useful for classification.

As a case study, we use data from the long-base stereovision Bioseco BPS [10]. The raw data from the system are preprocessed, and then the basic features, such as 3D positions, velocity, distance, and object size, are extracted. In order to reduce the number of features, the basic features are fused, e.g., size/distance or velocity/distance. All features are normalised using statistical representations such as histograms and the distribution parameters, including average value, variance, etc.

## 4. Data Preparation and Feature Preselection

The data handling process of the applied classification system consists of two stages. The first step is related to data acquisition and preprocessing. Then, preselected features are extracted and normalised from enhanced data.

### 4.1. Data Gathering and Preprocessing

**Figure 2:**
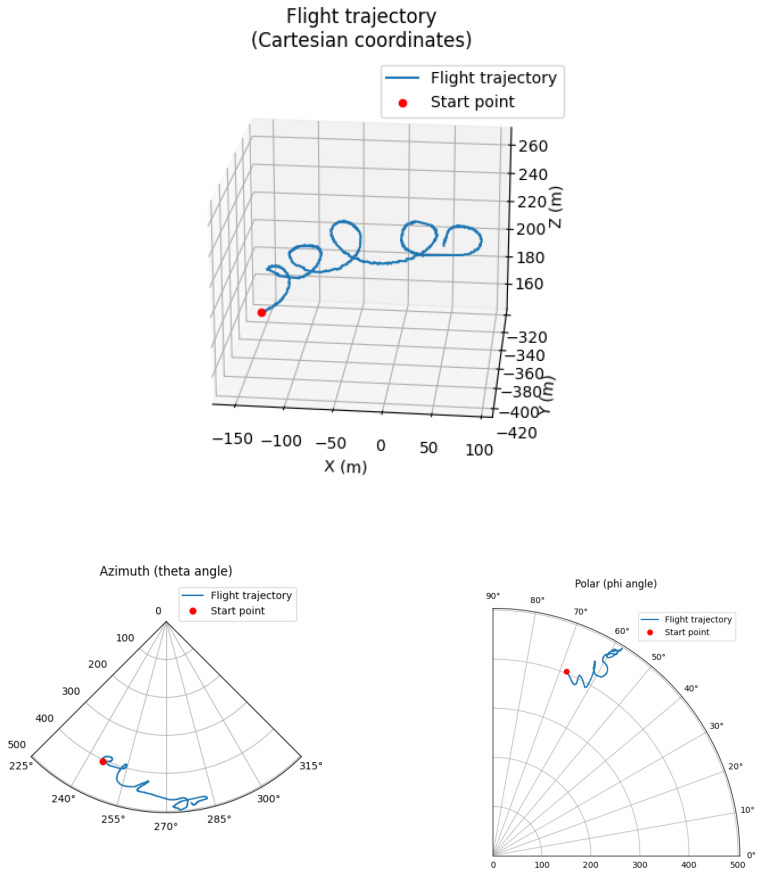
A white-tailed eagle’s flight trajectory, with a typical soaring pattern in Cartesian co-ordinates and two projections of spherical co-ordinates.

The test data used for feature extraction originate from the BPS system. By processing this data, birds’ flight trajectories can be defined. An example of flight trajectory in Cartesian and spherical co-ordinate systems is presented in Figure 2.

The database used comes from BPS installed at one of the wind farms located in Northern Germany. Each record includes the co-ordinates of a 3D flight path, 4k video, and cropped images of the detected objects [31]. A complete long-range BPS comprises eight detection modules installed around a wind turbine tower. Each module is equipped with four cameras coupled in two pairs of stereovision. The system simultaneously detects and localises multiple objects in the observation zone [10].

The retrieved data of species labelled by experts involves a set of the following: *timestamp*, *image size*, and the co-ordinates of the object’s centre of gravity pixel position on the images of both cameras: [*x_top_camera*, *y_top _camera*], and [*x_bottom_camera*, *y_bottom_camera*]. Pixel positions are denoised by Friedman’s super smoother [32] and were used to estimate an object’s spatial position [*x, y, z*], as in [10]. However, in this work, spherical co-ordinates (phi, theta, radius) are used to reduce the propagation of quantisation uncertainty of distance estimation [7,10].

For monitoring purposes, all detected items are divided into reports/paths and labelled by ornithologists into three classes of objects: *aeroplanes*, *big raptors*, and *other birds*. The birds of prey we were interested in are *Accipitriformes*—raptors with a wingspan over 1.1 m, such as *Buteo buteo*, *Milvus milvus*, *Haliaeetus albicilla*.

### 4.2. Features Extraction and Normalisation

In order to reduce the system’s computational complexity without compromising accuracy, only the features relevant to the classification task must be selected and extracted from the database. In this study, the parameters were chosen in such a way that they are descriptive enough to capture an object’s behaviour but are also easy to compute. There are four physical characteristics extracted from the data of flight trajectory, which form the base of the features:

1. *Polar angle*, φ, of object localisation in spherical co-ordinates, in radian.
2. Object *angular velocity*, calculated for the polar and azimuth angles, $\omega_{\varphi }$ and $\omega_{\theta }$, respectively, in radian/second.
3. Object *size* extracted from the image, calculated as the number of an object’s pixels, $size_{px}$, in pixels.
4. *Arc path lengths*, calculated for polar and azimuth angles, $arc_{\varphi }$ and $arc_{\theta }$, respectively, as the path lengths in the polar and azimuth spherical co-ordinates, in meters.

All these features, apart from object *size*, are defined in spherical co-ordinates because of their robustness and resistance to possible distortions, allowing for a reduction in the propagation of quantisation uncertainty in distance estimation. However, despite these measures, quantisation errors in distance measurements by a stereovision system can still cause even large objects detected from afar to be inaccurately localised in closer proximity and falsely labelled as medium-sized, potentially creating problems in distinguishing aeroplanes from birds.

**Figure 3:**
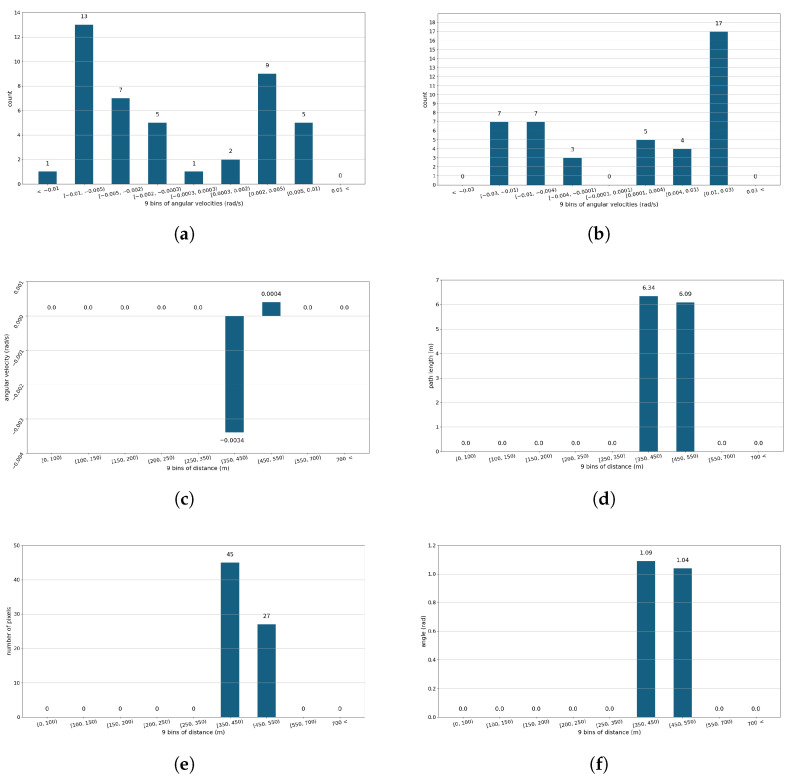
An example of feature extraction from preprocessed data, based on flight trajectory from Figure 2. (**a**) Histogram for angular velocity $\omega_{\varphi }$. (**b**) Histogram for angular velocity $\omega_{\theta }$. (**c**) Average angular velocity $\omega_{\varphi }$ per distance. (**d**) Average arc path lengths in $arc_{\varphi }$ per distance. (**e**) Average number of image pixels ($size_{px}$) of the detected object per distance. (**f**) Average polar angle φ per distance.

The $\omega_{\varphi }$, $\omega_{\theta }$, $arc_{\varphi }$, and $arc_{\theta }$ are measured within a 2-s window, while the φ and $size_{px}$ are sampled at 1/16 s. The reduction of computational complexity can be achieved by structuring the data from the monitoring system. It can be carried out by using the statistical properties of the data and relating them to relevant variables, e.g., distance. In Table 1, the features defined in three columns represent the following structures:

1. The *histograms* of the *angular velocities* $\omega_{\varphi }$ and $\omega_{\theta }$ depict the characteristics of the detected object’s flight. The histograms use nine bins for $\omega_{\varphi }$〈−0.01, 0.01〉 and $\omega_{\theta }$〈−0.03,0.03〉 values; see Figure 3a,b.
2. The bar diagram of *average angular velocities* for distance intervals. The features depict changes in flight characteristics with distance, e.g., angular velocities are lower at large distances; see Figure 3c.
3. The bar diagram of *average polar angle*, $\bar{\varphi }$, with respect to distance intervals; see Figure 3f.
4. The bar diagram of *average size*, $\bar{size_{px}}$, concerning distance intervals, which strongly depend on the distance, grows as the distance decreases; see Figure 3e.
5. The bar diagram of *average arc path lengths*, $\bar{arc_{\varphi }}$, and $\bar{arc_{\theta }}$; see Figure 3e.
6. The variances of each physical quantity were computed to depict their statistical characteristics.

Therefore, for each classification stage, six feature groups based on four physical quantities are used. If we apply nine-bin histograms and nine-bin average value structures relative to nine distance intervals, then 78 numerical variables are used. However, because of extreme values in the dataset, *z-score normalisation* has to be used for all variance parameters and most of the average values relative to distance intervals. Furthermore, the values for $size_{px}$ are logarithmised and min-max normalised. The histograms of $\omega_{\varphi }$ and $\omega_{\theta }$ are processed as percentages.

## 5. Optimal Feature Selection for Classification

Two feature selection methods have been evaluated: the correlation coefficients filter (CCF) and GAs. The features are then used by DT, RF, NN, and SVM classifiers for object recognition. In the following subsections, we explain the methods used, and then the results are presented.

### 5.1. Ga- and CCF-Based Optimal Feature Selection Methodology

The experiments were performed in Python 3.10 with the aid of scikit-learn v.1.2.0 package [33], which includes all mentioned classifiers. Some configurations of the classifiers’ hyperparameters were tested in a few preliminary experiments, but these adjustments did not improve the classification scores. Therefore, default settings on scikit-learn classifiers were used, which can be viewed in the online documentation. For example, by default, RF uses 100 trees with no maximal depth restrictions and Gini impurity as a splitting measure.

Moreover, some modifications within these classifiers were also considered, and the class threshold could be adjusted according to the following:

(1) $$predict\_class\left(sample\right)=\left\{\begin{array}{cc} 1, & \;\mathrm{if}\;\;probability(1|sample)\geq threshold,\\ 0, & \;\mathrm{otherwise}. \end{array}\right.$$

Binary classifiers calculate the probability of both classes (predict_proba method in scikit-learn), and the sample admits the class with the higher probability, so the default threshold is 0.5. Lowering the threshold gives more weight to class 1, thus ensuring that it is more frequently detected. For example, if the sample had the following predicted class probabilities: (aeroplane/0:0.55,bird/1:0.45) and the threshold is set to 0.4, then the observation has a ’bird’ class assigned. It must be noted that this method itself is not involved in the training process and is only used for testing.

**Figure 4:**
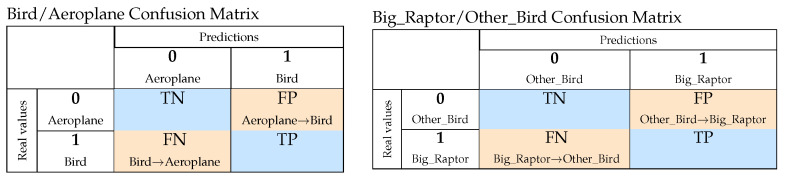
Confusion matrices for classification tasks where True_Negative = TN, True_Positive = TP, False_Positive = FP, and False_Negative = FN.

The dataset is checked for class balance, and it is modified if a significant class bias is detected. The training set contains 75% randomly chosen samples from the dataset. The remaining samples are used for validation. The confusion matrix is computed for all the classification models, as shown in Figure 4. Two measures are used to evaluate their performance: *accuracy* and *recall*, as defined by Equation (2). *Accuracy* prescribes the overall performance quality in terms of the percentage of correct predictions. *Recall* describes the probability of a correct positive answer. If the positive class is considered more important, then maximising *recall* ensures that we have fewer false negative detections (FN). This is crucial in this study, which prioritises nature conservation over energy production efficiency. False negatives occur when a bird is misclassified as an aeroplane or a big raptor is misclassified as a common bird. This leads to the potential danger of a wind turbine not stopping and a fatal clash with an endangered bird; this risk should be minimised. On the other hand, false positives (false alarms) stop the wind turbine unnecessarily, leading to energy production loss. This error is, however, of lesser concern.

(2) $$Accuracy=\frac{TP+TN}{TP+FP+TN+FN},\;Recall=\frac{TP}{TP+FN}$$

The first method used for feature selection is based on a correlation matrix. Pearson’s correlation coefficients between each of the 78 feature variables and binary classes are computed. Assuming that the features with correlations close to zero are the least discriminative, the feature selection algorithm works as follows:

1. Select *n* features with the highest absolute correlation coefficient to the class;
2. Use the selected features to train and evaluate a classifier five times with differently split training and test sets;
3. Return the average *accuracy* and *recall* on the test set from the five iterations.

This CCF was run for all the investigated classifiers, and the best global result was chosen.

The second method of feature selection is a GA, which is based on and developed by using the Python PyGAD package (v. 3.3.1) [34]. The GA is classifier-dependent; therefore, each classifier must be used separately as a fitness evaluator.

GA-based feature selections are encoded as binary chromosomes of length equal to the number of variables, which, in our case, is 78. A gene 1 in the chromosome is a flag that selects the feature, and a gene 0 is a flag discarding the feature. The population size is 400, with 200 *parents* chosen using a tournament selection of size 5. The *offspring* are created with a single point crossover and a 2.5% chance mutation, and only the *offspring* are added to the new population. The algorithm runs for between 50 and 70 generations. The single fitness measure is calculated and returned. The algorithm can be described as follows:

- Set minimal thresholds $th_{a}$ and $th_{r}$ for the *accuracy* and *recall*, respectively. These thresholds define our goal performance. For the Aeroplane/Bird classification, they are set to 95 and 97, respectively, and for Big_Raptor/Other_Bird classification, they are 75 and 90, respectively, as is stated in Section 3.
- Use the thresholds to define the two variables: modified accuracy $A=100\times Accuracy-th_{a}$, and modified recall $R\;=\;100\times \;Recall\;-\;th_{r}$.
- Use *A* and *R* to define Acc_Component and Rec_Component, as shown in (3), (4). If *A* or *R* is negative, Acc_Component or Rec_Component admit negative values that decrease rapidly, decreasing the *accuracy* and *recall* to reach below the goal thresholds.
  (3) $$Acc\_Component=\left\{\begin{array}{cc} A, & \;\mathrm{if}\;\;A\geq 0,\\ -1.3\cdot {\left|A\right|}^{\frac{3}{2}}, & \;\mathrm{if}\;\;A<0. \end{array}\right.$$
  (4) $$Rec\_Component=\left\{\begin{array}{cc} R, & \;\mathrm{if}\;\;R\geq 0,\\ -1.3\cdot {\left|R\right|}^{\frac{3}{2}}, & \;\mathrm{if}\;\;R<0. \end{array}\right.$$
- Calculate the fitness value from the following:
  (5) Fitness_Value=Acc_Component+Rec_Component

Firstly, the classification performance was evaluated using the correlation method and all four classifiers with different class thresholds were included. For the Aeroplane vs. Bird classification, 11 thresholds were considered: 0, 0.1,⋯, 1.0. For the Big_Raptor vs. Common_Bird task, the thresholds were selected with higher decimal precision. For both classifiers, the best ones with optimally chosen thresholds were chosen and then used as fitness evaluators in the GA feature selection method. The results for best classifiers with optimal thresholds are then displayed for both selection methods. The features chosen for classification in the case of both methods and both tasks are also presented.

**Figure 5:**
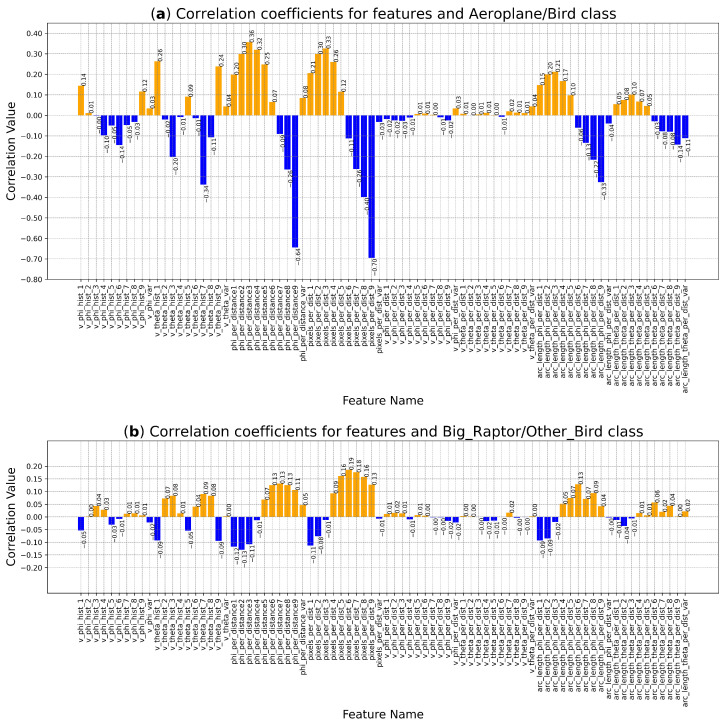
Correlation coefficients for numerical features: (**a**) the Aeroplane/Bird classes in the whole dataset; (**b**) the Big_Raptor/Other_Bird classes in the bird-only dataset. Positive correlation: orange, negative correlation: blue. Note: the scales on the *y* axis are different for both charts.

Correlation coefficients for both classification tasks are presented in Figure 5. It is worth noting that the values for the Aeroplane/Bird dataset are moderately high, meaning that distinguishing aeroplanes from birds should be more accurate. The correlation coefficients for the Big_Raptor/Other_Bird classification are relatively low, indicating that using them for classification may result in low scores.

### 5.2. Validation of the Optimal Feature Selection for Different Classifiers

The dataset used for validation comprises 3536 aeroplanes and 8748 birds, including 1325 big raptors. Both classification tasks, *Aeroplane/Bird* and *Rare/Common Birds*, were balanced in manageable proportions, 1:2.5 and 1:5.5, respectively, and did not require balancing techniques.

Classifiers for each classification task were trained and tested for different numbers of features selected by the CCF. In the case of Aeroplane/Bird classification, the overall performance of all classifiers is very high. All classifiers with default thresholds have an *accuracy* of over 90% and a *recall* of over 95% if we consider at least the 12-highest correlated features. However, the RF-based classifier outperforms other classifiers from 1% to 3%. It is the only classifier that has reached the desired *accuracy* and *recall* goal performance of 95% and 97%, as stated in Section 3. After changing the threshold from the default value of 0.5 to a new value of 0.4, *recall* increases by 0.6–0.7%, and the required performance goal is still met, so we admit the adjusted value for further study. For 60 features, the RF classifier with th=0.4 reached the peak performance of an *accuracy* and *recall* of 95.7% and 98.6%, respectively.

For the Big_Raptor vs. Other_Bird classification, all models except RF yielded poor or moderately good results. The NN performed best for 20–30 features, with the *accuracy* and *recall* ratios ranging from 62% to 80% for lower thresholds of 0.075 and 68% to 74% for higher thresholds of 0.125. The DT failed to define a good compromise between the two measures, returning 45% and 83% for most feature selections and thresholds. SVM worked best with around 70 selected features, with the most balanced result of 63% and 73% for a threshold of 0.125. Minimal changes in threshold led to very imbalanced results, such as *accuracy* = 27% and *recall* = 95% or *accuracy* = 80% and *recall* = 51%.

**Figure 6:**
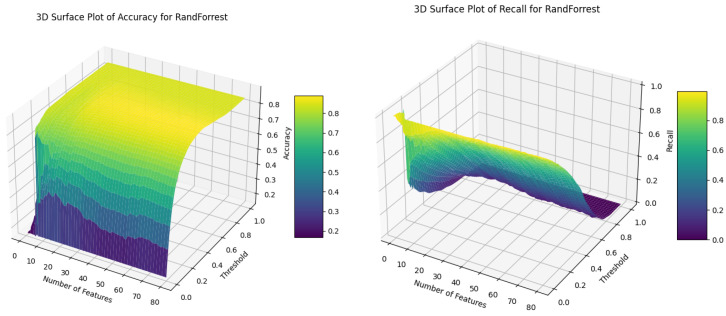
Big_Raptor/Other_Bird classification performance of RF; accuracy and recall scores for the number of features and thresholds.

**Figure 7:**
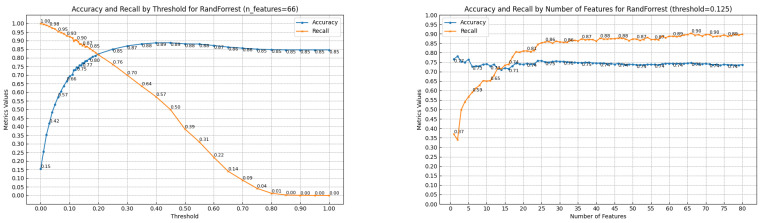
Big_Raptor/Other_Bird classification performance of RF; accuracy and recall scores for a fixed number of features (66 (**left**)) and for a fixed threshold (0.125 (**right**)).

RF performs best, reaching an *accuracy* and *recall* ratio of 75% and 90%, respectively, for 66 features at a threshold of 0.125. With higher thresholds between 0.18 and 0.2, its performance is more balanced, ranging from *accuracy* = 80% and *recall* = 85% to *accuracy* = 82% and *recall* = 82%. The scores plotted as surfaces with respect to the number of features and thresholds are presented in Figure 6. Line plots for the metrics values vs. threshold for 66 features and the metrics values vs. the number of features for threshold 0.125 can be seen in Figure 7. From the plots, one can see how selecting an optimal threshold is crucial for obtaining high classification scores, especially for *recall*, which is more sensitive to the thresholds than *accuracy*. For clarification, *recall*’s variability comes from the fact that it relies on minority classes (big raptors represent only 15% of all the birds), while *accuracy* depends on the whole dataset and is more stable.

The second feature selection approach using the GA is classifier-reliant because a certain classifier is used as a fitness evaluator. Therefore, we only employ this method for the best two classifiers:

- RF (th=0.4) for Aeroplane/Bird classification;
- RF (th=0.125) for Big_Raptor/Other_Bird Classification.

This experiment investigated whether the performance scores could be improved for some other feature subset not indicated by CCF. The GA runs twice for 50 to 70 generations for both classification tasks, and the outputs with better results were chosen. The scores from the first generation of GA already outperformed those from CCF. The *accuracy* and *recall* measures stabilise after about 10 generations of a quick increase. The features selected for the Aeroplane/Bird classification resulted in only a 1.3% increase in *accuracy*, with no change to *recall* compared to CCF. The features selected for the Big_Raptor/Other_Bird classification resulted in a substantial increase in *recall* of 3.1%. The complete results are shown in Table 2.

**Figure 8:**
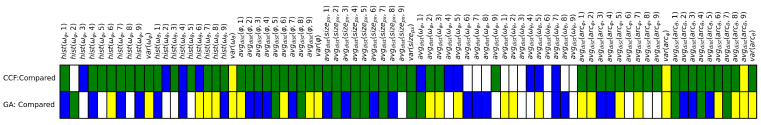
Optimal feature selections for both binary classification tasks, Aeroplane/Bird and Big_Raptor/Other_Bird, and two selection methods: CCF and GA. In green 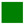: features selected for both classification tasks by the given selection methods; In yellow 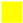: features selected only for the Aeroplane/Bird classification; In blue 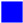: features selected only for hte Big_Raptor/Other_Bird classification task; In white: nonselected features.

In Figure 8, the feature sets from the CCF and GA selection methods for both binary classification tasks using an RF-based classifier are presented. It can be seen that GA selects fewer features, and they are more discriminative for each classification task, while for the correlation method, most of the selected features are the same for both tasks.

## 6. Discussion

An attempt has been made to develop a methodology for optimal feature selection for flying object classification. The results were sufficient enough to consider implementing the system in challenging real-time applications such as bird protection on wind farms or airports. A cascade classification approach with GA feature selection and RF classifiers allowed for the distinction of endangered birds of prey from common birds, with a recall of 93.5%, and birds from other flying objects, with a recall of 98.6%, while still maintaining a high accuracy of 77.2% and 97%, respectively.

Like other tree-based classifiers, the random forest algorithm is quick and is suitable for real-time applications. However, RF can only parse numerical or categorical data, not images or videos. Therefore, in future research, features extracted from images using convolutional NNs, and videos or trajectories with recurrent NNs, transformers, or other deep-learning models could be applied. More advanced machine learning methods involving cost-sensitive training and loss function manipulation could yield even better results.

In the scope of the research, we quantitatively evaluated the contribution of different features provided by the Bioseco BPS to enhance classification performance. Features such as histogram-like structures coupled in different distance ranges allowed for the development of high-performance classification models. This idea could be used jointly with other 3D features, as mentioned in Section 2, such as turning angle, curvature, or acceleration, potentially leading to even more powerful models.

Feature selection and its impact on classifier scores were tested in this study. Two feature selection techniques were employed: simple correlation-based filtering and a more sophisticated wrapper-type GA method. Selecting features highly correlated with the class improved the scores only by a fraction of a percentage. However, the genetic algorithm was shown to be the most efficient selection tool, and the scores increased by up to 3% compared to scores with no feature selection.

The presented methodology helped to determine which features are most distinguishable and can be used to classify birds from aeroplanes and big raptors from other birds. The feature $avg_{dist}\left(size_{px},\cdot \right)$ was the most useful for both classification tasks. This indicates that the correct size estimation and object distance provided by the BPS are crucial for constructing reliable machine-learning-based classifiers. Additionally, Figure 8 reveals that using the correlation method for selection applies much less diversity of features. In contrast, the GA-based approach is most adaptable to the given tasks, and the selected features for both tasks do not overlap with each other.

However, GA is rather slow and stochastic in nature. Other wrapper-type approaches, such as recursive feature elimination (RFE), could be more efficient in feature selection and could be considered in future research.

Furthermore, a larger and more representative database, including additional records from different locations, is required to provide real-time performance for developed algorithms. The authors continue their co-operation with ornithologists to acquire more data.

Finally, the authors wish to mention that this study is part of a broader research project focusing on a collision risk assessment system. 

## Figures and Tables

**Table 1:** Pre-selected features, grouped by physical characteristics.

Quantity	Symbol	Physical Unit	Features		
			Histogram with *n*-th Distance Interval	Average Value within *n*-th Distance Intervals	Variance
**Angular velocity**	$\omega_{\varphi }$	$\frac{radian}{second}$	$hist(\omega_{\varphi },\Delta dist_{n})$	$avg_{\omega_{\varphi }}\left(\Delta dist_{n}\right)$	$var(\omega_{\varphi })$
	$\omega_{\theta }$		$hist(\omega_{\theta },\Delta dist_{n})$	$avg_{\omega_{\theta }}\left(\Delta dist_{n}\right)$	$var(\omega_{\theta })$
**Polar angle**	φ	radian		$avg_{\varphi }\left(\Delta dist_{n}\right)$	var(φ)
**Size**	$size_{px}$	pixel		$avg_{size_{px}}\left(\Delta dist_{n}\right)$	$var(size_{px})$
**Arc path length**	$arc_{\varphi }$	meter		$avg_{arc_{\varphi }}\left(\Delta dist_{n}\right)$	$var(arc_{\varphi })$
	$arc_{\theta }$			$avg_{arc_{\theta }}\left(\Delta dist_{n}\right)$	$var(arc_{\theta })$

**Table 2:** Performance metrics of RF-based two binary classifiers with the threshold of 0.4 and 0.125, for all features and two feature selection methods: CCF and GA.

	Airplanes vs. Birds (RF, *th* = 0.4)			Big_Raptors vs. Other_Birds (RF, *th* = 0.125)		
	Full Features	CCF	GA	Full Features	CCF	GA
	(78 Features)	(60 Features)	(39 Features)	(78 Features)	(66 Features)	(38 Features)
Accuracy	95.5%	95.7%	97%	73.7%	74.6%	77.2%
Recall	98.6 %	98.6%	98.6%	89.8%	90.4%	93.5%

## Data Availability

The presented data are accessible for authorized staff according to the local regulation.
